# The Clinical Effectiveness of School Screening Programme for Idiopathic Scoliosis in Malaysia

**DOI:** 10.5704/MOJ.1703.018

**Published:** 2017-03

**Authors:** AS Deepak, JY Ong, DSK Choon, CK Lee, CK Chiu, CYW Chan, MK Kwan

**Affiliations:** National Orthopaedic Center of Excellence for Research and Learning (NOCERAL), University of Malaya, Kuala Lumpur, Malaysia; * Department of Orthopaedic Surgery, Prince Court Medical Center, Kuala Lumpur, Malaysia

**Keywords:** adolescent idiopathic scoliosis, school screening, prevalence, positive predictive value

## Abstract

**Introduction:**

There is no large population size study on school screening for scoliosis in Malaysia. This study is aimed to determine the prevalence rate and positive predictive value (PPV) of screening programme for adolescent idiopathic scoliosis.

**Materials and Methods:**

A total of 8966 voluntary school students aged 13-15 years old were recruited for scoliosis screening. Screening was done by measuring the angle of trunk rotation (ATR) on forward bending test (FBT) using a scoliometer. ATR of 5 degrees or more was considered positive. Positively screened students had standard radiographs done for measurement of the Cobb angle. Cobb angle of >10° was used to diagnose scoliosis. The percentage of radiological assessment referral, prevalence rate and PPV of scoliosis were then calculated.

**Results:**

Percentage of radiological assessment referral (ATR >5°) was 4.2% (182/4381) for male and 5.0% (228/4585) for female. Only 38.0% of those with ATR >5° presented for further radiological assessment. The adjusted prevalence rate was 2.55% for Cobb angle >10°, 0.59% for >20° and 0.12% for >40°. The PPV is 55.8% for Cobb angle >10°, 12.8% for >20° and 2.6% for > 40°.

**Conclusions:**

This is the largest study of school scoliosis screening in Malaysia. The prevalence rate of scoliosis was 2.55%. The positive predictive value was 55.8%, which is adequate to suggest that the school scoliosis screening programme did play a role in early detection of scoliosis. However, a cost effectiveness analysis will be needed to firmly determine its efficacy.

## Introduction

Scoliosis is a three-dimensional deformity of the spine defined as a lateral curvature of the spine in the coronal plane with a Cobb angle more than 10 degrees. Idiopathic scoliosis had the prevalence of about 0.4% to 7% amongst adolescents in Asian countries ^1^-^12^. In patients with idiopathic scoliosis, earlier detection and diagnosis allows early conservative treatment, which is bracing and this can avoid unnecessary surgery and preserve a higher health-related quality of life scores^13^, ^14^. Late detection may lead to higher rates of patient needing surgery and if this condition is left untreated, it can progress to severe scoliosis, which has been shown to affect pulmonary function of patients^14^, ^15^. Moreover, the severe scoliotic deformity will also affect patients’ self-image and this may leave an irreversible psychological impact on this group of patients.

A school screening programme can detect students with early deformity. The percentage of students diagnosed of scoliosis amongst those positively screened is known as the positive predictive value (PPV). Amongst the Asian countries, the PPV of school scoliosis screening ranged from 20% to 70%^1^, ^3^, ^4^, ^6^, ^9^-^11^. A previous study done in Malaysia has reported a PPV school scoliosis screening of 35.7% with a small study population of 832 students^6^. Therefore, we aimed to report the prevalence rate and the PPV based on a larger series of school screening for adolescent scoliosis in Malaysia.

## Materials and Methods

This was a cross sectional screening programme carried out in schools (11 schools) which agreed to participate in Kuala Langat, Selangor, Malaysia from August 1996 to April 1999. This region had a population of a mixture of rural and urban people. Based on previous prevalence studies^16^, ^17^, we have recruited students aged between 13 and 15 years old from 11 secondary schools. A total of 8966 students (4381 males and 4585 females) were recruited.

**Fig. 1:**
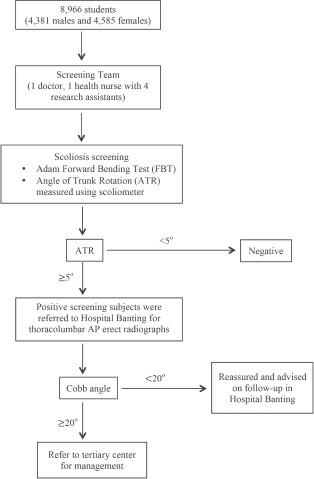
Screening protocol of Adolescent Idiopathic Scoliosis in Kuala Langat, Malaysia.

The school screening team consisted of a doctor, a health nurse and four research assistants. All the volunteered students were examined with Adam forward bending test at first. The angle of trunk rotation (ATR) was then measured at thoracic, thoracolumbar and lumbar region by using a scoliometer if the Adam forward bending test detected any asymmetry of the trunk. ATR of 5 degrees or more was considered positive. These positive screened students were referred to the nearest district hospital, Hospital Banting for anteroposterior standing whole-spine radiograph. Cobb angle would be measured. Those with Cobb angle of 20 degrees or more would be referred to the scoliosis clinic at the tertiary centre, University Malaya Medical Center for further assessment and management. The rest of the subjects were reassured and advised on follow-up (Fig. 1).

For radiological assessment, we used Cobb angle of >10 degrees as suggested by Scoliosis Research Society to diagnose scoliosis. Based on the Cobb angle measured from the radiographs, the prevalence rate was calculated. The positive predictive value (PPV), which is the percentage of students diagnosed of scoliosis amongst those positively screened, which denotes a measure of clinical effectiveness, was also calculated. The prevalence rate and PPV analysis were further divided into three categories according to the Cobb angle i.e. >10 degrees, >20 degrees and >40 degrees).

## Results

In this screening programme, 8966 students were screened using scoliometer. There were 410 students screened positive i.e. ATR > 5 degrees in this programme but only 156 (38.0%) students turned up for the radiological assessment. Those who failed to attend the radiological examination were not included in the statistical analysis.

There was a higher referral rate of female students compared to male students (Table I). The overall referral rate of male students was 4.2% compared to 5.0% for female students. The referral rate for male students rose from 3.5% for 13-year-old students, 4.0% for 14-year-old students to 5.2% for 15-year-old students. The female students recorded almost similar referral rate for 13-year-old (5.0%), 14-year-old (4.9%) and 15-year-old (5.0%).

**Table I:** Referral rate for radiographs using a scoliometer screening

Age	13		14		15		13-15		Total
Gender	M	F	M	F	M	F	M	F	
Population screened	1636	1644	1469	1477	1276	1464	4381	4585	8966
Positive subjects	57	82	59	73	66	73	182	228	410
Referral rate (%)	3.5	5.0	4.0	4.9	5.2	5.0	4.2	5.0	4.6

M = Male, F = Female

The prevalence rate is 0.97% for Cobb angle more than 10 degrees, 0.22% for more than 20 degrees and 0.04% for more than 40 degrees. Taking the drop-out rate of 62% into consideration, the adjusted prevalence rate is 2.55% for Cobb angle more than 10 degrees, 0.59% for more than 20 degrees and 0.12% for more than 40 degrees. The adjusted prevalence rate is 2.55%, 0.59% and 0.12% for Cobb angle >10°, >20° and >40° respectively (Table II).

**Table II:** Prevalence rate and positive predictive value

Cobb angle	Prevalence		^*^Adjusted prevalence rate (%)	PPV	
	Rate (%)	95% CI		Rate (%)	95% CI
>10°	87/8966 (0.97)	0.0079, 0.0120	2.55	87/156 (55.8)	0.635, 0.477
>20°	20/8966 (0.22)	0.0014, 0.0034	0.59	20/156 (12.8)	0.154, 0.101
>40°	4/8966 (0.04)	0.0002, 0.0011	0.12	4/156 (2.6)	0.0382, 0.0129

PPV = Positive predictive value

* Adjusted prevalence rate = prevalence rate/turn-up rate x 100%; turn-up rate=38%

Based on the 156 positive screened students who turned up, the positive predictive value (the percentage of students diagnosed of scoliosis amongst those positively screened using scoliometer) was 55.8% for Cobb angle more than 10 degrees, 12.8% for more than 20 degrees and 2.6% for more than 40 degrees (Table II).

## Discussion

Screening is defined as ‘the presumptive identification of unrecognised disease or defect by application of tests, examination or procedures which can be applied rapidly’^18^. In adopting this definition, the commission on chronic illness further stated that ‘screening tests sort out apparently well persons who have a disease from those who probably do not’. A screening test is not intended to be diagnostic. Persons with positive or suspicious findings must refer to their physicians for further diagnosis and treatment. Scoliosis screening fits into this definition because it is able to detect those with true scoliosis from those without and refer them for subsequent diagnostic test to facilitate early intervention, thus decreasing need for surgery through bracing.

The first scoliosis screening programme was started in Delaware, USA in the late 1950s and subsequently scoliosis screenings were carried out elsewhere, either by legislation or voluntarily^1^, ^16^, ^19^-^23^. The implementation of school scoliosis screening was debatable over decades as there were large variations across studies in term of its PPV. The variations were likely due to the diversity in study design, referral criteria, screening tests used, frequency of screening and duration of follow-up^24^. However, Ohrt-Nissen *et al*^25^ confirmed in their study that referred patients by general practitioner from schools without screening programme had a larger curve magnitude compared to patient from schools with a scoliosis screening programme.

The impact of scoliosis screening on the reduction of surgical treatment was described in several studies. Lonstein *et al*^26^ had screen a quarter of a million children for eight years and 3.4% were referred for evaluation and 1.2% were found to have scoliosis. Since the initiation of the school screening, the number of children requiring surgical procedure diminished and the average curve for those who had surgery reduced from 60 degrees to 42 degrees. Montgomery and Willner^27^ had found that the demand for surgery reduced from 45% to 10% in the screening group of patients and concluded that bracing outcome was better in the screening group because of the earlier onset of intervention. Bunge *et al*^28^ found that there was no evidence that screening for scoliosis reduced the need for surgery. This may be due to the controversies they had in the effectiveness of early treatment with bracing and students screened positive might not had brace treatment initiated immediately.

In 2013, the BrAIST clinical trial confirmed the effectiveness of bracing in AIS. This was a multicenter prospective study with enrollment of 242 patients. 116 patients were randomized to either bracing or observation whereas 126 chose between bracing or observation. When both randomized and preference cohorts were analyzed the treatment success was 72% after bracing compared to 48% after observation. In the intention to treat analysis, the rate of treatment success was 75% among patients randomly assigned to bracing compared to 42% among those randomly assigned to observation. The trial was terminated earlier due to the clear advantage of bracing in arresting progression in AIS^29^.

When it comes to national screening programme, cost effectiveness analysis plays an important factor to drive its implementation. Lonstein *et al*^26^ found that the cost of a school screening programme for scoliosis was low and it was a cost effective measure which should be carried out. Montgomery *et al*^30^ further added that clinical screening using scoliometer that was combined with Moire screening would further improve the cost effectiveness of the programme. Soucacos *et al*^31^ found that the cost of screening process was negligible compared to the benefit of decreased number of operative procedures performed after the screening programme, the identification of a large number of previously undiagnosed curves which were subsequently treated with operation or brace, and the identification of children who were at high risk of progression. Thilagaratnam *et al*^32^ found that school based scoliosis screening programme was cost effective and the effectiveness can be improved further by targeting the screening at high risk groups, such as prepubertal females. However, Morais *et al*^16^ found that the screening cost per child was high in Canada and mass screening for idiopathic scoliosis was not justified. This was supported by Yawn *et al*^33^ who found the school scoliosis screening was significantly more costly than previously reported.

The measurement of ATR using scoliometer was a noninvasive, radiation free and comparatively cheap method of screening. It can be easily implemented and it had been proven to have good correlation with radiological analysis (r=0.7, p<0.05) and very good intra-rater reliability^34^, ^35^. By using this screening method, we found that percentage of students referred for radiographs in the present study was 4.6%. Therefore, 1 in 20 students will be referred for further radiography. There were more females referred with the male to female ratio of 1:1.2. The prevalence rate of idiopathic scoliosis for this study, according to the definition of scoliosis with the Cobb angle >10 degrees, was 2.55%. This corresponds to the prevalence of idiopathic scoliosis in Asian countries that varies between 0.4% - 7%^1^-^12^ (Table III).

**Table III:** Literature reviews of school scoliosis screening in Asian countries

Year Published	Country	Programme	Cobb angle (degrees)	Sample size	Age (years)	Gender	PR (%)	PPV (%)
2005	Singapore	Wong *et al*^1^	>10	72,699	6 - 7	Male	0.02	17.6
						Female	0.05	23.7
					9 - 10	Male	0.15	24.5
						Female	0.24	24.1
					11 - 12	Male	0.21	23.8
						Female	1.37	50.1
					13 - 14	Male	0.66	27.5
						Female	2.22	47.7
2009	Singapore	Yong *et al*^2^	>10	93626	9	Female	0.27	-
					10		0.64	
					11		1.58	
					12		2.22	
					13		2.49	
2010	Hong Kong	Luk *et al*^3^	>10	157,444	10 - 19	-	2.49	76.5
			>20				1.39	36.5
			>40				0.23	8.1
2011	Korea	Suh *et al*^4^	>10	1,134,890	10 - 14	Male	1.97	41.0
						Female	4.65	51.0
2011	Japan	Ueno *et al*^5^	>10	255,875	11 - 12	Male	0.04	-
						Female	0.78	
					13	Male	0.25	
						Female	2.51	
2013	Malaysia	Htwe *et al*^6^	>10	832	12	-	0.6	35.7
2014	Korea	Lee *et al*^7^	>10	37,856	11	Male	0.05	-
						Female	0.35	
2015	Hong Kong	Fong *et al*^8^	>10	306,144	5th G - 19	Male	2.2	81.0
						Female	4.8	
2015	Japan	Yamamoto *et al*	^9^ >10	195,149	5th G	Male	0.06	33.3
						Female	0.34	
					6th G	Male	0.01	
						Female	0.37	
					7th G	Male	0.06	
						Female	0.73	
2016	China	Du *et al*^10^	>10	6,824	6 - 17	Male	1.96	33.8
						Female	3.11	43.2
2016	China	Hengwei *et al*^11^	>10	99,695	10 - 19	-	5.14	-
2016	China	Zheng *et al*^12^	>10	11,024	10 - 11	Male	0.17	-
						Female	0.08	
					12 - 13	Male	0.52	
						Female	0.88	
-	Malaysia	Present Study	>10	8,966	13 - 15	-	2.55	55.8
			>20				0.59	12.8
			>40				0.12	2.6

PR = Prevalence rate, PPV = Positive predictive value, G = Grade

The PPV (the percentage of students diagnosed of scoliosis amongst those positively screened) for this study was 55.8% for students with a Cobb angle of more than 10 degrees. This finding was higher than previously reported PPV for scoliosis screening in Malaysia by Htwe *et al*^6^. We also found that our school screening programme was more predictive than other Asian country such as Singapore (27.5% - 47.7%)^1^, Korea (41.0% - 51.0%)^4^, China (33.8% -43.2%)^10^, and Japan (33.3%)^9^. Only the school screening programme done in Hong Kong^3^, ^8^ had a higher PPV than our study (76.5% - 81.0%). In their study, those negatively screened who had ATR between 0 - 2 had the tests repeated biennially, those negatively screened who had with ATR 3 -4 had the test repeated annually. And those with ATR between 5 and 14 or obvious signs of trunk or shoulder asymmetry were further evaluated by Moirè topography. These additional interventions were possibly the cause of the improvement in PPV in their school screening programme. Thus, with a PPV of 55.8%, we found that school programme can be a viable intervention to improve the detection and to employ early treatment of scoliosis in Malaysia.

A major limitation of this study was the dropout rate. There were 410 students screened positive in this programme but only 156 (38.0%) students turned up for the radiological assessment. This was a voluntary programme and the requirement for a radiographic evaluation was not compulsory. Furthermore, parents’ health awareness regarding scoliosis was low. This may explain the high dropout rate in this study. The method of sampling in this study may consist selection bias and hence not reflective the true prevalence and positive predictive value for Malaysia.

## Conclusion

The prevalence rate of scoliosis was 2.55% and scoliosis was more common amongst female. The positive predictive value, which reflects the percentage of students diagnosed of scoliosis amongst those positively screened using scoliometer, was 55.8%. This predictive value was adequate to suggest that the school screening programme did play a role in early detection of scoliosis. However, a cost effectiveness analysis will be needed in order to firmly determine its efficacy.
